# Improved compaction of ZnO nano-powder triggered by the presence of acetate and its effect on sintering

**DOI:** 10.1088/1468-6996/16/2/025008

**Published:** 2015-04-08

**Authors:** Benjamin Dargatz, Jesus Gonzalez-Julian, Olivier Guillon

**Affiliations:** Friedrich Schiller University of Jena, Otto Schott Institute of Materials Research, Löbdergraben 32, D-07743 Jena, Germany

**Keywords:** sintering, green body processing, nano-powder, zinc oxide, water, zinc acetate, zero radial shrinkage

## Abstract

The retention of nanocrystallinity in dense ceramic materials is still a challenge, even with the application of external pressure during sintering. The compaction behavior of high purity and acetate enriched zinc oxide (ZnO) nano-powders was investigated. It was found that acetate in combination with water plays a key role during the compaction into green bodies at moderate temperatures. Application of constant pressure resulted in a homogeneous green body with superior packing density (86% of theoretical value) at moderate temperature (85 °C) in the presence of water. In contrast, no improvement in density could be achieved if pure ZnO powder was used. This compaction behavior offers superior packing of the particles, resulting in a high relative density of the consolidated compact with negligible coarsening. Dissolution accompanying creep diffusion based matter transport is suggested to strongly support reorientation of ZnO particles towards densities beyond the theoretical limit for packing of ideal monosized spheres. Finally, the sintering trajectory reveals that grain growth is retarded compared to conventional processing up to 90% of theoretical density. Moreover, nearly no radial shrinkage was observed after sinter-forging for bodies performed with this advanced processing method.

## 1. Introduction

Zinc oxide (ZnO) is an n-type semiconductor that is extensively used in electronic and optoelectronic systems due to its combination of piezoelectric properties, gas reactivity and wide band gap of 3.3 eV [1]. ZnO is one of the most promising ceramics for challenging applications in the fields of ultrafast nanolasers, cost efficient solar cells, nanogenerators for self-powered devices and thin film gas sensors [2–6]. Nevertheless, prior to the expected outstanding final properties, understanding of the mechanisms involved in the coarsening of nanocrystalline ZnO is required as excessive grain growth leads to deterioration of properties and functionality. As most physical properties are affected by the residual porosity, it is essential to obtain nearly full densification. Unfortunately, the sintering of nanocrystalline, dense bulk ZnO with grain sizes <100 nm is still a challenging task. Roy *et al* [7] sintered nanocrystalline ZnO powder pellets, but the final grain size was 1.5 *μ*m. Mazaheri *et al* [8] fully densified ZnO and retarded grain growth by means of two-step sintering, but ended up with a mean grain size of 1.4 *μ*m. Hynes *et al* [9] attained grains with size approximately 500 nm but achieved only 95% of theoretical density (% TD) by hot pressing. In general, the retention of nanocrystallinity depends on the competition between densification and grain growth, the driving forces for which both depend on reciprocal grain size [10, 11]. The most promising approaches to suppress grain growth are pinning of grain boundaries (by the addition of a second phase), application of external pressure [8], very fast heating [12–14] or two-step sintering [8, 15]. However, particle dispersion and green body processing are key steps to achieve this goal. Thus, the whole processing chain needs to be considered beginning with a homogeneous and high green density sample prior to sintering at elevated temperature [10, 11]. The shaping of green bodies from nanocrystalline powders by dry pressing typically results in compacts containing agglomerates and 50–61% TD [8, 16, 17], although values of 73% TD [18] for cold isostatic pressing are reported. Alternative shaping technologies overcome the problems of dry pressing utilizing suspensions and benefit from lowered friction between the particles. Wet processing, e.g. gel casting, slip casting or pressure filtration, results in a homogeneous packing with typical green densities between 50 and 65% TD [10, 15, 19]. However, well-dispersed stable suspensions are required, which can be difficult to achieve with nanoparticles. This study presents a new approach for the processing of ZnO nanoparticle compacts resulting into homogeneous green bodies with an extremely high density.

## 2. Experimental procedures

### 2.1. Materials and methods

The standard ZnO powder utilized for the experiments is referred to as ZinCox10 (IBU-tec advanced materials AG, Weimar, Germany) with a purity of >99.00 wt% and mean particle size of 10 nm from the data sheet of the producer. An additional ZnO powder (NG20, Nanogate AG, Quierschied-Göttelborn, Germany) with a purity of >99.99 wt% and a primary particle size between 20 and 50 nm was used as the reference powder. Furthermore, a third powder was used for the compaction, which was produced by mixing the pure ZnO powder (NG20) with a total amount of 4.2 wt% zinc acetate dehydrate Zn(H_3_C–COO)_2_ (Sigma Aldrich, St. Louis, USA). A total amount of 0.4 g powder was stored in a glass beaker inside an environmental chamber (KBF 240, Binder GmbH, Tuttlingen, Germany). The storage conditions were defined by temperatures between 20 and 85 °C and absolute moistures between 1.0 and 140 g m^−3^. In particular, 85 °C with 140 g m^−3^ moisture (40% relative humidity), 85 °C with 1 g m^−3^ moisture (0.3% relative humidity) and 20 °C with 14 g m^−3^ moisture (85% relative humidity) will be further referred to ‘humid warm’, ‘dry warm’ and ‘humid cold’ conditions, respectively. The NG20, ZinCox10, and NG20 + acetate powders were each poured into a hard metal pressing die (5 mm diameter and 8.5 mm initial height), after storage of one hour under a particular environmental condition. Then, the ZnO powders were pressed either for 20 h within the environmental chamber or by conventional cold dry pressing for 1 min. In the former procedure the pressing tool was installed inside the environmental chamber under the same respective storage condition, and a uniaxial pressure of 50 MPa was applied for 20 h. The axial displacement of the metal punches was measured every second during the pressing step by a laser triangulation sensor (LK-G10, KEYENCE Corp., Osaka, Japan) with ±0.1 mm spatial resolution. The axial displacement was correlated to the change in density (as the powder is compressed in a stiff mold and as compaction takes place only vertically). In comparison, green bodies were produced with dried ZinCox10 powder by conventional dry cold pressing for 1 min at 50 MPa. Therefore, ZinCox10 powder had been dried for 24 h prior to the pressing step by storing in a drying cabinet at 105 °C with ≪0.5 g m^−3^ moisture (≪0.1% relative humidity). The pressed ZnO bodies were ejected after the pressing step and installed in the sinter-forging device for further compaction (see next section).

### 2.2. Sintering study

Green bodies of humid warm and conventionally processed ZinCox10 powder were sintered in air. First, samples were heated up to 400 °C for 20 min isothermal time with a rate of 5 K min^−1^ in order to burn out residual organic content. The samples were further heated to 700 °C with a rate of 20 K min^−1^ and an isothermal time of one hour. Afterwards, the sintered sample was cooled down with 10 K min^−1^ to room temperature. The furnace for sintering was a modified sinter-forging setup, which is composed of a vertical split furnace fixed on a mechanical testing machine (Model 5565, Instron Corp., Norwood, USA). The samples were sintered freely or under an external pressure of 3, 15 and 40 MPa. The actual height and diameter of the samples were measured *in situ* by a laser system (162-100; BETA Laser Mike Inc., Dayton, USA) every 2.5 s (by averaging 128 values) with a resolution of 0.5 *μ*m. Thus, the true strain during sintering could be calculated. The densification curves were calibrated by the density of sintered specimens measured at room temperatures by the Archimedes method.

### 2.3. Characterization

Microstructure of compacted and sintered ZnO bodies was investigated by transmission electron microscopy (TEM) and by high resolution scanning electron microscopy (HRSEM). TEM was performed with a JEM-3010 (JEOL, Akishima, Japan) operated at 300 kV. HRSEM was performed with a field emission microscope Auriga60 (Carl Zeiss AG, Oberkochen, Germany). The grain size measurement was carried out on polished samples using the line interception method with the software ‘Lince’ (v. 2.31, Ceramics Group, TU Darmstadt) using a factor of 1.56, evaluating at least 300 grains [20]. Phases for the powder and compacted specimens were characterized by means of x-ray diffraction (XRD). XRD measurement was carried out using a D8-Discover (Bruker AXS, Billerica, USA) with Cu–K_*α*_ radiation at *λ* = 1.540 56 Å, operated at 40 kV and 40 mA, a step size of 0.02° and a counting time of 1.6 s. Crystallite sizes were determined using the Scherrer equation after fitting the (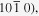 (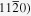 and (0002) Bragg peaks with the pseudo-Voigt function. A shape factor *K* = 0.94 was applied for the Scherrer equation [21] and full width at half maximum was corrected for instrumental broadening. Fourier-transform infrared spectroscopy (FTIR) measurements were carried out with an ‘Alpha’ spectrometer (Bruker Optics, Billerica, USA) using the absolute total reflection method. The pressed samples and the raw material zinc acetate were ground up before FTIR measurements. Each measurement was performed twice with approximately 20 mg powder and a resolution of 4 cm^−1^. Thermogravimetric analysis was performed with a ‘DTA/TG-92’ (SETARAM Instrumentation, Lyon, France) on 5 mg dried ZnO powder which was heated up to 750 °C in a platinum container at a rate of 10 K min^−1^.

## 3. Results and discussion

### 3.1. Analysis of untreated ZnO powder

**Figure 1.**
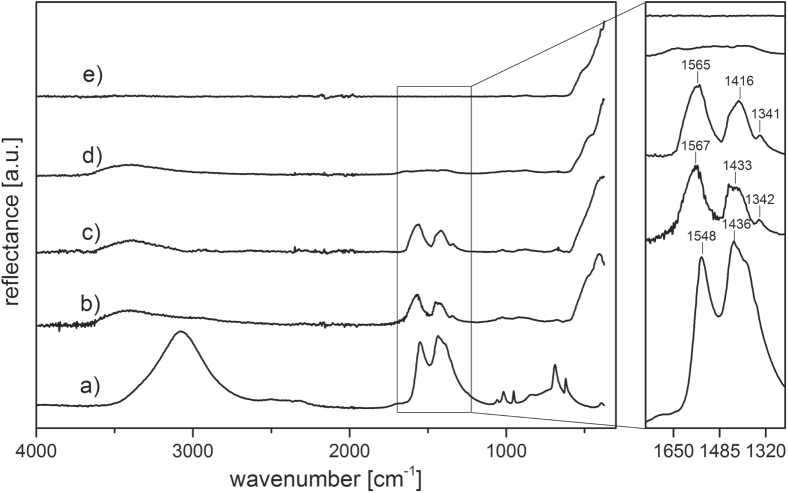
FTIR spectra of (a) zinc acetate dihydrate as-received non-compacted powder, (b) as-received non-compacted ZinCox10, (c) ZinCox10 powder compacted 20 h under humid warm condition (85 °C, 140 g m^−3^ moisture) (d) as-received non-compacted NG20 powder and (e) ZinCox10 powder fired at 800 °C. The spectra are offset for clarity.

The particle size of both commercial powders was investigated prior to compaction study. The high purity powder (NG20) showed a mean particle size of 26 ± 9 and 33 nm for TEM and XRD observation, respectively. The standard powder (ZinCox10) exhibited a mean particle size of 16 ± 9 and 17 nm for TEM and XRD investigation, respectively. Thus, the mean crystallite size given by XRD corresponds to the particle size observed by TEM and is about twice as large for NG20 powder compared to ZinCox10 powder. A previous study [22] proved by TEM and XRD that the ZnO particles are isometric. Further, TEM confirms the presence of isometric particles with polyhedral shape. XRD analysis on both powders confirmed that all Bragg reflexes are attributed to the hexagonal wurtzite phase of ZnO. ZinCox10 powder was studied for its compaction and sintering behavior. A previous investigation [22] confirmed the presence of organic content (zinc acetate) inside the ZinCox10 powder by comparative infrared spectroscopy investigation with acetate dihydrate powder, whereas the coarser NG20 powder showed negligible impurity. The presence of zinc acetate is proven by FTIR analysis, which is of major interest for the explanation of the compaction behavior of the ZnO powder. FTIR spectra in reflectance mode are illustrated in figure 1 for zinc acetate dihydrate, NG20 and ZinCox10 powders. The broad band reaching from 2700 to 3600 cm^−1^ is present for all ZnO samples except for the sintered ZnO and corresponds to adsorbed water and OH groups [23]. The band at 470 cm^−1^ is found for all the ZnO specimens and can be attributed to the vibrational stretching mode of Zn–O bounding [24] (figures 1(b)–d)). The two bands around 1420 and 1560 cm^−1^ correspond to the symmetrical and asymmetrical stretching vibrations of carboxylate group, respectively [23, 25]. These bands are found for zinc acetate dihydrate powder as well as for the as received and the humid warm processed ZinCox10 powder. Furthermore, no acetate is present in the NG20 powder or after sintering of the ZinCox10 powder. Thus, the zinc acetate is only present in the ZinCox10 prior to sintering. It seems conclusive that residual zinc acetate is still present in the ZinCox10 powder, as zinc acetate is typically used as precursor for the synthesis of ZnO [26–30]. Thermogravimetric analysis of the dried ZinCox10 powder reveals a mass loss of 3.3 ± 0.2 wt% resulting from water and organic removal. If the entire mass is correlated to the molar amount of carbon present in acetate salt, approximately 4.16 wt% of zinc acetate should be contained in the ZinCox10 powder. Spitz *et al* [31] showed that acetate is covering the surface of ZnO particles. The theoretical thickness of a hypothetical continuous zinc acetate layer covering ZnO nanocrystals has been calculated by assuming a TD of 1.74 g cm^−3^ as well as ideally spherical ZnO nanoparticles with a mean diameter of 17.2 nm and a TD of 5.606 g cm^−3^. Such a homogeneous coating of zinc acetate is estimated to be 0.37 ± 0.02 nm thick, which is difficult to highlight by TEM for example.

### 3.2. Processing of ZnO green body

#### 3.2.1. Compaction behavior

**Figure 2.**
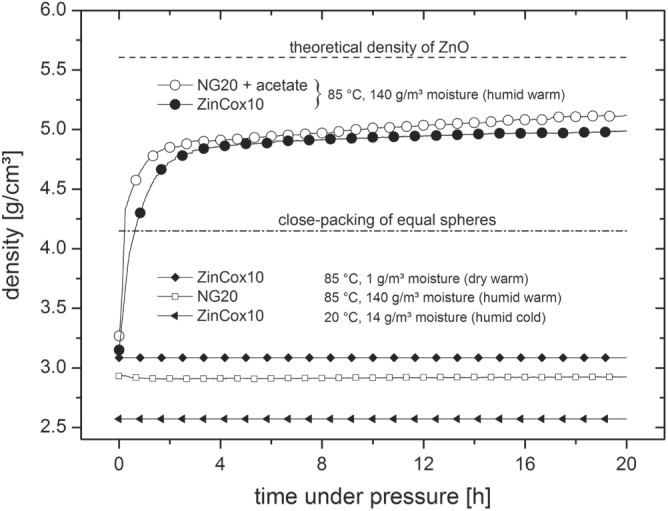
Density as a function of time for bodies under constant uniaxial pressure of 50 MPa under defined temperature and humidity. The dashed and dashed dotted lines give the theoretical density of pure ZnO and the density for close-packing of equal spheres, respectively. The compaction curves are represented by one measured data point each second.

The two nanocrystalline ZnO powders were investigated with respect to their compaction behavior. The absolute density is given as a function of time during compaction in figure 2. Interestingly, the initial density was around 3 g cm^−3^ (55% TD) at the beginning of compaction for both ZnO powders at 85 °C. In contrast, the compact under humid cold condition shows a constant density of 2.57 g cm^−3^ (45.8% TD). The pure NG20 powder was pressed to a density of 2.93 g cm^−3^ (52.3% TD) under humid warm condition and shows no further increase in density with time. Densities around 45–57% TD for green compacts are typical even for dry, cold pressing at 50 MPa [10, 16, 32]. In contrast, the density of the ZinCox10 powder strongly increases within the first hour up to a value of approximately 4 g cm^−3^ if compacted under humid warm condition. The densification rate further decreases successively until a constant density of approximately 4.99 g cm^−3^ is achieved after 20 h of compaction. The measured absolute density of ZinCox10 powder compacts after compaction needs to be corrected in order to take into account the presence of acetate. The corrected density is 85.9% TD, which is extremely high. The addition of zinc acetate to pure NG20 powder results into the same compaction behavior under humid warm condition. To our knowledge, this study presents the highest reported value by uniaxial pressing of ceramic powders. Mazaheri *et al* [8] achieved 61% TD by cold uniaxial pressing at 200 MPa for pure ZnO powder with comparable initial nanoparticle size. Ewsuk *et al* [33] achieved similar green density with 62% TD by granulation, uniaxial and isostatic pressing at 140 MPa for both, macroscopic and nanoscale powder. Alternative methods, e.g. pressure filtration, slip casting of ceramic slurries, result into a density of 50–70% TD [10, 15, 19]. The slightly elevated temperature of 85 °C may raise the reorientation process and the presence of water may lower the friction between the ZnO particles. However, these findings are in contradiction with the model for the highest packing of ideal monosized spheres, whereas the packing density is theoretically restricted to 74% TD [34]. On top of that, particles were found to show polyhedral instead of spherical shape, which should result in an even lower maximum theoretical value. Thus, not solely reorientation, but another process discussed in the next section seems responsible for the huge gain in green density. In contrast, no change in density was measured during the compaction step neither under dry warm condition (85 °C, 1 g m^−3^ moisture) nor under humid cold condition (20 °C, 14 g m^−3^ moisture). Thus, a combination of temperature and humidity is required to increase the green body density, if acetate is present in the compacted ZnO powder.

#### 3.2.2. Microstructural analysis

**Figure 3.**
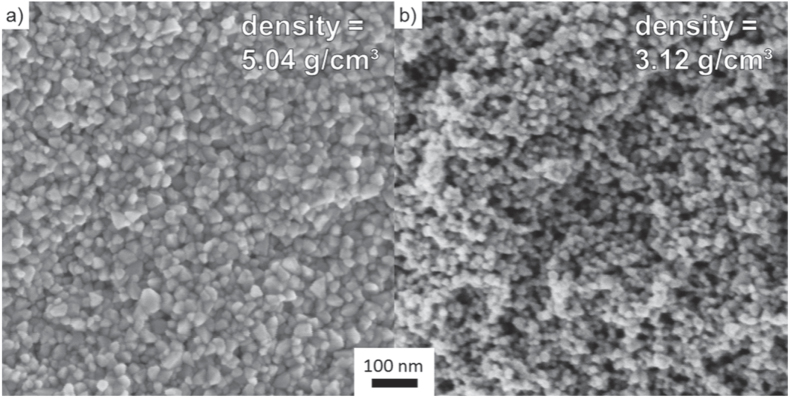
Fracture surfaces of ZinCox10 powder compacted 20 h under (a) humid warm condition (85 °C, 140 g m^−3^ moisture) and (b) dry warm condition (85 °C, 1 g m^−3^ moisture).

**Figure 4.**
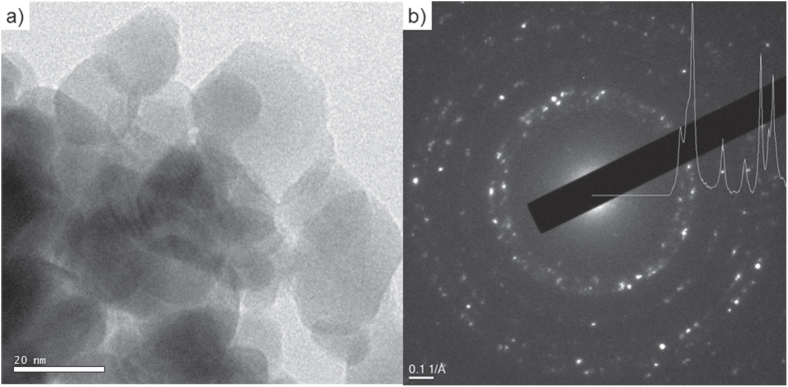
TEM investigation of ZinCox10 powder compacted 20 h under humid warm condition (85 °C, 140 g m^−3^ moisture) with (a) microstructure and (b) diffraction pattern.

**Figure 5.**
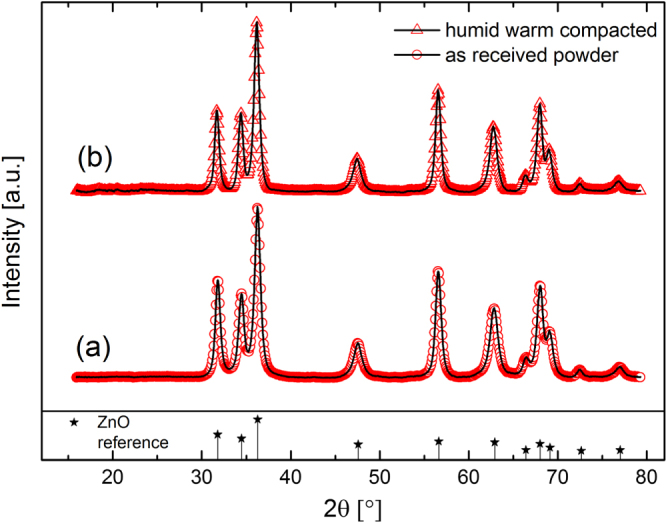
XRD pattern of ZinCox10 for (a) as-received non-compacted powder and (b) compacted powder after 20 h under humid warm condition (85 °C, 140 g m^−3^ moisture). The Bragg reflections for ZnO wurtzite structure [51] are represented below the measured data by stars. The measured data are offset for clarity and only one out of four data points is given.

Figure 3 shows SEM micrographs of fracture surfaces of samples, which were compacted in an environmental chamber under humid warm (85 °C, 140 g m^−3^ moisture) and dry warm (85 °C, 1 g m^−3^ moisture) conditions. The sample kept under the humid warm condition shows a regular distribution of particles and only small pores are found. This finding indicates a homogeneous and high packing of the ZnO crystallites (figure 3(a)). The median particle size was estimated from the fracture surface by SEM and it increased to 25 ± 7 nm (growth factor of 1.51 compared to the initial state) under the humid warm condition. In comparison, the SEM micrograph of the dry compacted sample shows a more heterogeneous particle packing and reveals the presence of pores in the scale of 10–100 nm (figure 3(b)). However, under the dry warm condition the mean particle size stays constant, while the absolute density remains constantly low. A former investigation [22] showed that massive coarsening of ZinCox10 particles takes place under the same environmental conditions, if the powder is stored for long time. Thus, preferential crystal growth along *c*-axis higher than 400% was observed within 24 h of storage. In contrast, the present study shows that particle coarsening is strongly retarded and no preferred crystal growth occurs if the powder is simultaneously compacted under 50 MPa, although the contacts between particles are fostered by pressure. This surprising compaction behavior only occurs for the ZinCox10 powder, but not for the NG20 powder. Therefore, the presence of zinc acetate in the ZnO powder is the key trigger for better compaction. Figure 4 shows TEM images from a sample compacted for 20 h under the humid warm condition (85 °C, 140 g m^−3^ moisture). The TEM micrograph in figure 4(a) gives an impression of grown ZnO particles. No indication was found for neck formation or grain boundaries between the ZnO nanocrystals by TEM and HRTEM investigation. The median primary crystallite size of 26 ± 8 nm confirms the results from SEM measurements (this represents an increase in crystallite size of 1.61 times). Figure 4(b) shows the TEM diffraction pattern with the intensity as a function of diffraction angle. The position of reflexes confirms the presence of the wurtzite phase and gives no hint for other phases. XRD analysis also gives no evidence for any additional phase except for the zincite phase of ZnO (figure 5). Neither TEM diffraction pattern nor XRD revealed the presence of phases other than wurtzite ZnO modification (figure 4(b)). The absence of possible additional phases by TEM analysis might be explained by a local temperature increase of the sample under the electron beam and zinc acetate might already be removed, as temperatures about 100 °C initiate strong degradation [35].

**Figure 6.**
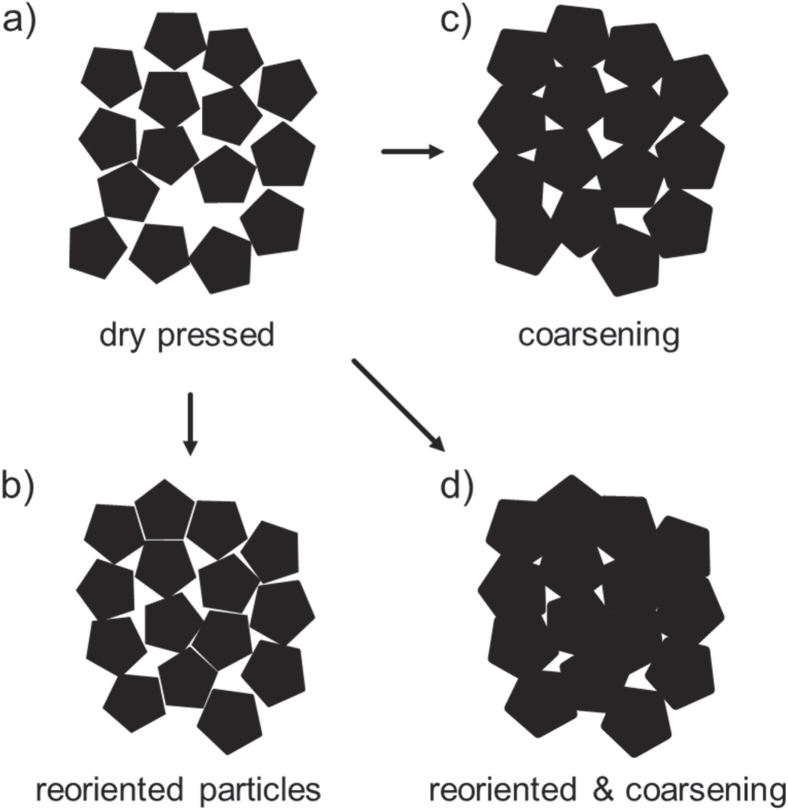
Schematic of the compaction process for polyhedral particles. Dry pressed particles for (a) as-received non-compacted powder, (b) after coarsening, (c) after reorientation and (d) combination of reorientation and coarsening.

On the other hand, a small nanosized layer of acetate would hardly be detected by HRTEM [36]. In general, there are two possibilities for how mass transport is taking part towards consequent reduction of pore volume under compaction without the necessary activation temperature for sintering. The lowest sintering temperature found for sintering of ZnO is reported to be 0.2 × *T*_m_ at an external pressure of 50 MPa [37], i.e. 400 °C, which is well above the temperature found in the environmental chamber. However, the threshold temperature for diffusion of zinc interstitials is below an absolute temperature of 130 K [38]. Moreover, this diffusion process was suggested for coarsening of ZnO particles at room temperature [39]. On top of that, acetate ion interaction with the ZnO particle surface should be taken into account. Thus, dissolved acetate ions can result into the formation of surface defects or enhance dissolution of the particle surface, as pH value should be decreased. As a consequence, the dissolution is resulting in an enrichment of dissolved zinc species, surrounding the ZnO particles. Thus, the aqueous phase can be assumed to be enriched with zinc species and hydroxide ions. Such an aqueous layer is enabled to flow between ZnO particle interspaces and precipitate again. Recently it was reported [25, 35] that formation of ZnO occurred by thermal degradation of zinc acetate at temperatures of 85 °C. Figure 6 illustrates the compaction process for polyhedral shaped ZnO particles beginning with dry pressed state (figure 6(a)). The application of higher external pressure breaks agglomerates and leads to reorientation process (figure 6(b)). Aqueous layer with dissolved zinc species is pulled between particle contacts by capillary forces at the same time. Effective contact area can be decreased by the coarsening of particles (figure 6(c)).

The resulting effect is shown in figure 6(d) via combination of reorientation and coarsening. In addition, activated solid state diffusion based matter transport could be activated by pressure resulting into further densification. Coble [40] described the grain boundary diffusion controlled creep process for polycrystalline materials. Under stress, grain boundaries act as sources and sinks for vacancies under tensile and compressive stress, respectively. Here, dissolved zinc ions can additionally interact with sinks for vacancies under compressive stress and further participate in the overall compaction process. However, neither grain boundaries nor particle necks were found to indicate a diffusive sintering process. The absence of grain boundaries seems to be conclusive, as the sintering temperatures of ZnO for free sintering are reported between 700 and 1200 °C [41–43].

### 3.3. Solid state sintering

#### 3.3.1. Pressureless densification

**Figure 7.**
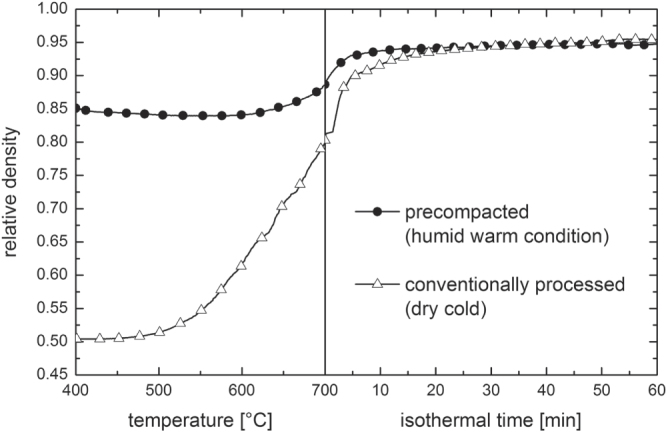
Densification curve for pressureless sintered ZnO bodies processed under humid warm and conventional conditions. Relative density is given as a function of temperature and isothermal time at 700 °C.

**Figure 8.**
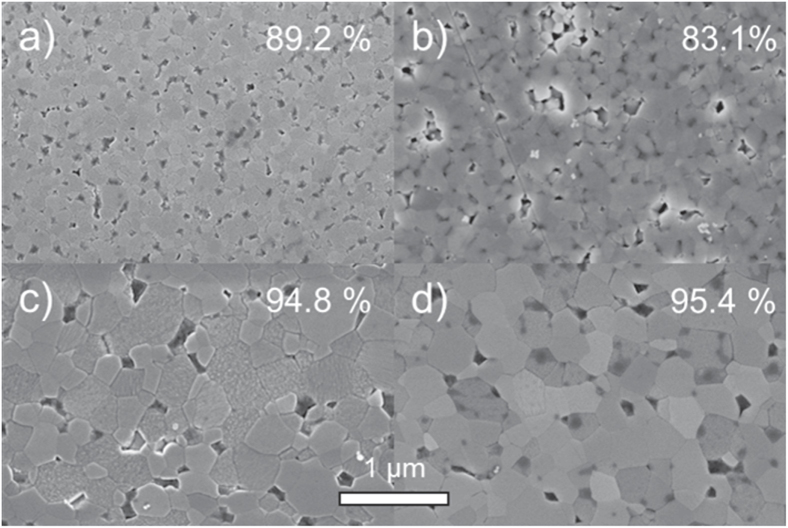
SEM images of polished surfaces for pressureless sintered ZnO samples for (a) and (c) humid warm compacted and (b), (d) conventionally processed green bodies. The samples were sintered at 700 °C (a), (b) without dwell and (c), (d) with dwell of 1 h. The determined relative density is labeled for each image in the right upper corner.

**Figure 9.**
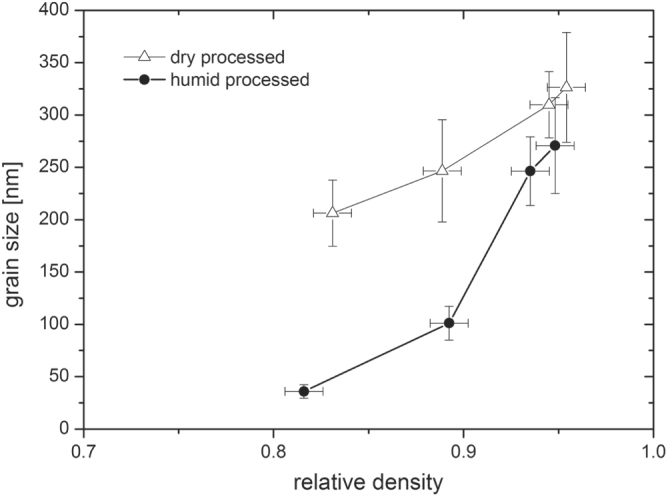
Sintering trajectory for ZnO bodies processed under humid warm and dry conditions and freely sintered.

The sintering of humid warm compacted ZnO samples seems promising in comparison to conventionally processed samples, as packing density is already very high prior to sintering step. Thus, a modified sintering trajectory is expected which should be shifted towards smaller grain sizes at a given density compared to dry processed green bodies. The densification behavior for freely sintered ZnO is illustrated in figure 7. The relative density is plotted as a function of temperature (during the heating stage) and isothermal time at a maximum temperature of 700 °C. The specimens start with 50 and 85% TD during initial stage of sintering for dry and humid warm conditions, respectively. Besides, the onset of densification with a temperature of 600 °C is 120 °C above the specimen processed under dry condition. Thus, sinterability of humid warm processed ZnO is decreased, as surface energy for humid warm specimen is reduced due to coarsening of particle size by 50% during green body processing. In comparison, particle size remains constant under conventional processing giving a slightly lower initial particle size prior to sintering. Nevertheless, maximum density of 95% was found to be equal for both samples after one hour at 700 °C, although this value was achieved 10 min later for conventionally processed specimen (dry condition) during isothermal dwell time. Hynes *et al* [9] observed similar final density for pressureless sintering of nanocrystalline ZnO powder at 700 °C. Additionally, Hynes *et al* reported about abnormal grain growth with grain sizes ranging from 67 to >530 nm, which is in contrast to the present study. The microstructural development of sintered ZinCox10 specimens is illustrated in figure 8. In general, no abnormal grain growth was observed. Figures 8(a) and (b) show microstructures that were obtained by an interrupted heating experiment for specimen processed under humid warm and dry conditions, respectively. The mean grain size with *d*_50_ = 101 nm is much smaller for humid warm processed specimen compared to conventionally processed ZnO (*d*_50_ = 206 nm), although density is already 6% higher. On the other hand grain size (*d*_50_ ∼ 300 nm) and density are similar for both specimens after sintering one hour at 700 °C (figures 8(c) and (d)). This grain size is surprisingly low taking into account that no external pressure was applied during sintering. In comparison, Mazaheri *et al* [8] hot pressed ZnO with equal initial powder particle size under 50 MPa external pressure and achieved a significant higher grain size (>600 nm) for the same density. The sintering trajectory (grain size versus relative density) is given in figure 9 with values that were obtained by interrupted heating experiments by sintering schedule from figure 7. The grain sizes of sintered specimen with conventionally processed ZnO result into more than two times higher values compared to humid warm processing for relative densities below 90%. The grain size increases rapidly for >90% TD, until similar grain sizes are achieved for both processing routes around 95% TD.

#### 3.3.2. Pressure-assisted densification

**Figure 10.**
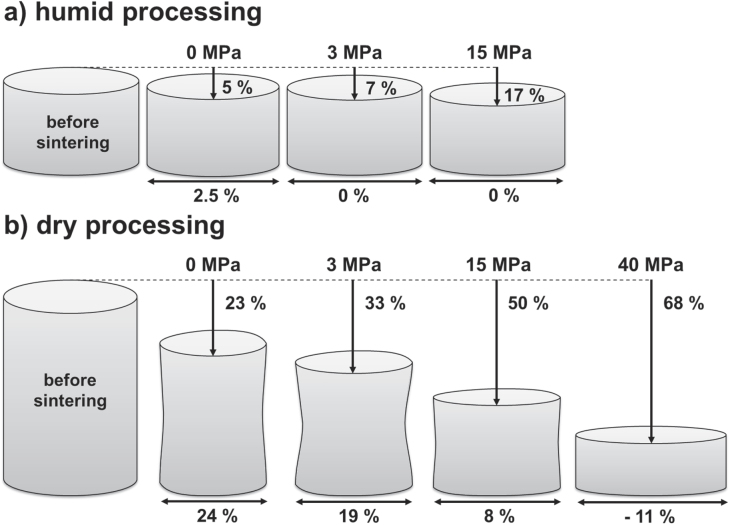
Shape evolution of compacted ZnO bodies before and after firing at 700 °C for 1 h for (a) humid and (b) dry processed green bodies. Axial and radial shrinkage are labeled for each applied external pressure.

In the following, a uniaxial pressure of 15 or 40 MPa was applied during sintering. Table 1 lists the obtained respective grain sizes and relative densities for humid warm and dry processed specimens. The final density achieves 95% TD, when an external pressure of 15 MPa is applied, but grain size increases as well to 600 nm. Dry processed samples show a comparable behavior. Here, density is stagnating around 95.5% TD and grain size is increasing up to 1.4 *μ*m. The evolution of final relative density is within 0.2%, which lies within the error range. Thus, density is not affected by the application of an external pressure, whereas the grain size is increasing with increasing external pressure. Interestingly, the grain size is smaller for the dry condition than for humid warm processed bodies at a uniaxial pressure of 15 MPa. Typically, the application of an external pressure retards grain growth in comparison to free sintering of ceramics, as the mechanism of densification is enhanced while the one of coarsening is not affected. Nevertheless, similar results are found in the literature on sintering of alumina [44, 45] and BaLa_4_Ti_4_O_15_ [46, 47]. Besson and Abouaf [44, 45] sintered pure alumina by hot isostatic pressing and found a final grain size proportional to the applied stress (up to 200 MPa). They suggested that the applied stress induced point defects and dislocations during densification, which are eliminated by grain growth. Amaral *et al* [46, 47] observed exaggerated grain growth during densification of sintered constrained films of BaLa_4_Ti_4_O_15_, which resulted in a much larger grain size for a given density than for freely sintered bulk. Interestingly, the grain size of dry processed ZnO samples remains lower compared to the humid warm condition at an external pressure of 15 MPa (table 1). High strain rates are suggested at the beginning of the sintering in order to eliminate interagglomerate pores prior to the onset of the final stage of sintering, when substantial grain growth occurs [48]. It is already known that neck growth and densification are promoted in the plane perpendicular to its direction under uniaxial loading which may also lead to anisotropy of a sintering body [49]. Figure 10 schematically illustrates the shape evolution of sintered samples from pressureless sintering towards increasing applied external pressure, which corresponds to axial (*∊*_z_) and radial (*∊*_r_) strain. Only 2.5% radial strain was detected for the pressureless sintered specimen under humid warm processing (figure 10(a)). Under dry processing, radial and axial strains are nearly equal (24%) for pressureless sintering, which was already reported before for free sintering (figure 10(b)) [18]. Thus, strains in both directions are equal (axial versus radial strain ratio *∊*_z_/*∊*_r_ ∼ 1), whereas a strain ratio *∊*_z_/*∊*_r_ of 2.5 was found for the humid warm specimen, but with absolute strain nearly one order of magnitude lower than under the dry condition. In general, the evolution of the strain ratio strongly depends on the applied external pressure. Rahaman *et al* [18] have already reported for sintered ZnO in 1991, that the strain ratio *∊*_z_/*∊*_r_ increases from ∼1 to ∼1.56 by the application of a uniaxial pressure. Moreover, they found that the strain ratio *∊*_z_/*∊*_r_ regains with a value of ∼1.2 by a strong increase in initial green density (73% TD). Thus, an increase of initial green density will decrease the radial stain and accordingly a lower pressure is required for zero radial strain. This finding is convenient with the results of the present study, whereas the strain ratio is lower for humid warm processed ZnO (∼5 g cm^−3^ initial density) in comparison to the dry condition (∼3 g cm^−3^ initial density). In addition the surface of the dry sintered samples shows a concave curvature. On the one hand, the degree of curvature is decreasing with increasing pressure, which is conclusive. On top of that, the radial strain is negative (−11%) under a uniaxial pressure of 40 MPa for dry processed specimens, resulting in a radial expansion, whereas similarly high pressures of 40–100 MPa would lead to a radial expansion for the humid warm specimen as well. Finally, zero radial shrinkage was already achieved by the application of a uniaxial pressure of 3 MPa. In the case of the dry processed specimen, linear interpolation of radial strain data from figure 10 gave a uniaxial pressure of 27 MPa for zero radial strain. It seems conclusive that the higher initial density of the humid warm processed specimen governs a lower sintering stress in axial and radial directions. Bordia *et al* [50] showed that a compressive uniaxial pressure between 20 MPa and 30 MPa during the intermediate stage of sintering is necessary to achieve a zero radial strain rate for sintering submicron alumina. This value, which depends on the viscous parameters of the sintering material, is in good agreement with the presented results of dry processed ZnO in this study.

**Table 1.** ZnO specimen sintered one hour at 700 °C for external applied pressures between 0 and 40 MPa. The table lists achieved relative density and grain size for specimens processed under humid warm and conventional conditions.

Green body processing	Applied pressure (MPa)	Relative density (%)	Grain size (nm)
	0	94.8	271
Humid warm	15	95.0	600
	0	95.4	326
Dry	15	95.4	342
	40	95.5	1419

## 4. Conclusions

The presented shaping methodology results in green compacts with densities superior to those obtainable by dry or standard wet shaping processes. In general, this new compaction behavior of ZnO offers a promising approach for retaining nanocrystallinity due to a strongly increased green density prior to the sintering step. Acetate was identified to activate this advanced shaping methodology for ZnO green bodies, if compacted under uniaxial pressure in presence of moisture and very low temperature. In contrast, pure ZnO showed no further compaction, but subsequent addition of acetate to pure ZnO powder results in high packing density in the same way. A final density of 95% TD was achieved not only for the humid processing, but also for the dry processing. In addition, nearly zero radial shrinkage was observed without the application of an external pressure. In contrast, an external pressure of 27 MPa is necessary to avoid radial shrinkage by means of conventional processing. Retention of grain growth is superior for densities lower than 92% TD for the presented shaping methodology. The burnout of the organic component hinders densification at high densities. Thus, a retarded grain growth is expected if the amount of acetate is properly decreased in order to decrease the influence of trapped gases. Further, it is likely that compaction and growth behavior of other metal oxide nanocrystals, synthesized on the basis of acetate or even other organic precursors, is similarly enhanced. However, further experiments with other metal oxides are required to validate the above mentioned role of acetate combined with water and temperature.
